# Supplementary material for: MetaQTL: a package of new computational methods for the meta-analysis of QTL mapping experiments
Source: BMC Bioinformatics. 2007 Feb 8;8:49. doi: 10.1186/1471-2105-8-49 (PMC1808479; doi:10.1186/1471-2105-8-49)
Supplement: Additional File 7 — MetaQTL Package : jar file and tutorial. This Zip archive contains both the MetaQTL JAR file and the files of the tutorial. [file 1471-2105-8-49-S7.zip › org.inra.metaqtl/doc/org/metaqtl/factory/TreeFactory.html]

TreeFactory


|  |  |  |
| --- | --- | --- |
| **Overview** \| **Package** \| **Class** \| **Use** \| **Tree** \| **Deprecated** \| **Index** \| **Help** | |  |
| **PREV CLASS**   NEXT CLASS | **FRAMES**    **NO FRAMES**     **All Classes** |  |
| SUMMARY: NESTED \| FIELD \| CONSTR \| METHOD | DETAIL: FIELD \| CONSTR \| METHOD |  |


---


## org.metaqtl.factory Class TreeFactory

```
java.lang.Object
  org.metaqtl.factory.TreeFactory
```

---

``` public final class TreeFactory extends java.lang.Object ```

A factory to deal with distance trees.

---

**Constructor Summary**

`TreeFactory()`


| **Method Summary** | |
| --- | --- |
| `static Tree` | `read_newick(java.io.Reader reader)` |
| `static Tree` | `read_newick(java.lang.String treeStr)` |
| `static Tree` | `read(java.io.Reader reader)` |
| `static Tree` | `read(java.lang.String str)` |
| `static void` | `write(Tree tree, java.io.OutputStream stream)`             This method writes out the given tree `nodes` as an array of `TreeNode` on the given output stream stream. |
| `static void` | `write(Tree tree, java.io.PrintWriter writer)`             This method writes out the given tree `nodes` as an array of `TreeNode` using the given writer writer. |

**Methods inherited from class java.lang.Object**

`clone, equals, finalize, getClass, hashCode, notify, notifyAll, toString, wait, wait, wait`

**Constructor Detail**

### TreeFactory

```
public TreeFactory()
```


**Method Detail**

### write

```
public static void write(Tree tree,
                         java.io.OutputStream stream)
                  throws java.io.IOException
```

:   This method writes out the given tree `nodes` as an array of
    `TreeNode` on the given output stream stream. The
    user is responsible for closing the stream.

    :   **Parameters:**: `nodes` - the tree: `stream` - the output stream. **Throws:**: `java.io.IOException`

---


### write

```
public static void write(Tree tree,
                         java.io.PrintWriter writer)
```

:   This method writes out the given tree `nodes` as an array of
    `TreeNode` using the given writer writer.

    :   **Parameters:**: `nodes` - the tree: `writer` - the writer.

---


### read\_newick

```
public static Tree read_newick(java.io.Reader reader)
                        throws java.io.IOException
```

:   **Parameters:**: `cur` -: `root` -: `reader` - **Throws:**: `java.io.IOException`

---


### read\_newick

```
public static Tree read_newick(java.lang.String treeStr)
                        throws java.io.IOException
```

:   **Parameters:**: `treeStr` - **Returns:**: **Throws:**: `java.io.IOException`

---


### read

```
public static Tree read(java.io.Reader reader)
                 throws java.io.IOException
```

:   **Parameters:**: `buffer` - **Returns:**: **Throws:**: `java.io.IOException`

---


### read

```
public static Tree read(java.lang.String str)
                 throws java.io.IOException
```

:   **Throws:**: `java.io.IOException`


---


|  |  |  |
| --- | --- | --- |
| **Overview** \| **Package** \| **Class** \| **Use** \| **Tree** \| **Deprecated** \| **Index** \| **Help** | |  |
| **PREV CLASS**   NEXT CLASS | **FRAMES**    **NO FRAMES**     **All Classes** |  |
| SUMMARY: NESTED \| FIELD \| CONSTR \| METHOD | DETAIL: FIELD \| CONSTR \| METHOD |  |


---
